# Editorial: Innovative approaches in veterinary pathology: diagnostics, therapeutics, and zoonotic threats

**DOI:** 10.3389/fvets.2026.1784641

**Published:** 2026-02-09

**Authors:** Gabriele Rossi, Francisco J. Salguero

**Affiliations:** 1 School of Veterinary Medicine, Murdoch University, Murdoch, WA, Australia; 2 United Kingdom Health Security Agency (UKHSA) Porton Down, Salisbury, United Kingdom

**Keywords:** animal welfare, antimicrobial resistance, CKD, copeptin, diagnostic technologies, pathogenesis

Veterinary Experimental and Diagnostic Pathology has undergone remarkable transformation in recent years, driven by advances in molecular biology (1), imaging technologies (2), and computational tools (3). This discipline plays a pivotal role in understanding disease mechanisms in animals, developing accurate diagnostic methods, and improving therapeutic strategies. Despite these strides, significant gaps remain in our knowledge of disease pathogenesis, diagnostic precision, and treatment efficacy, particularly in the face of emerging diseases, zoonotic threats, and antimicrobial resistance (4).

The research collected under this topic aims to address these challenges by presenting innovative approaches and critical insights into advances in disease pathogenesis, innovations in diagnostic technologies, therapeutic strategies and welfare, and zoonotic threats and surveillance. The overarching objectives are to identify pressing questions in veterinary pathology, test new hypotheses, and explore novel diagnostic and therapeutic strategies that can enhance animal health and welfare.

In this Research Topic there are 15 papers covering the above-mentioned aspects.

## Advances in disease pathogenesis

Several studies delve into the molecular and cellular underpinnings of disease. For instance, research from Quintanilla et al. highlights the role of porcine salivary carbonic anhydrase VI in disease pathogenesis, suggesting its potential as a biomarker for gastrointestinal and respiratory disorders. Similarly, the investigation from Zapico et al. has identified different nucleotide-binding oligomerization domain (NOD)-like receptors expresses in bovine paratuberculosis where they play a role in the pathogenesis. That underscore the importance of innate immunity in determining disease outcomes, offering new targets for intervention.

Research into renal warm ischemia in rats (Sánchez-Lara et al.) demonstrated activation of the transglutaminase pathway in tubulointerstitial fibrosis, offering parallels to feline chronic kidney disease and identifying TG2 inhibition as a promising therapeutic avenue. Additionally, a review on arginine vasopressin and copeptin (Paulin et al.) advocates for the validation of copeptin-based assays in veterinary medicine, which could revolutionize the diagnosis of polyuria-polydipsia syndromes.

## Innovations in diagnostic technologies

Rapid and accurate diagnostics are essential for disease control. This Research Topic features cutting-edge molecular tools, such as singleplex and duplex PCR assays for hypervirulent Klebsiella pneumoniae (Wang et al.), which promise faster detection of high-risk pathogens. Another example is from Liu et al., which developed an indirect ELISA for bovine coronavirus using recombinant N protein, this assay demonstrated 94.8% concordance with commercial kits, offering a cost-effective and specific diagnostic option.

Advances in molecular technologies, such as the CRISPR-based assays and digital droplet PCR designed by Kardoudi et al. for the diagnosis of fowl adenoviruses exemplify the shift toward high-sensitivity, field-ready diagnostics. Non-invasive approaches also emerge in Siddique et al. studies, with bioelectrical impedance analysis (Siddique, Batchu, et al.) combined with machine learning demonstrating potential for classifying goats affected by haemonchosis, and in AI-enhanced hematocrit analysis (Siddique, Panda, et al.) offering rapid anemia detection in small ruminants.

## Therapeutic strategies and welfare

Improving animal welfare through preventive and therapeutic measures is another focal point. A study from Galosi et al. on bacterial bedding conditioners in broiler chickens shows promising results in reducing footpad dermatitis, a major welfare concern. In companion animals, case reports such as necrotizing E. coli pneumonia in dogs (Vazin et al.) and cardiac lymphoma in cats (Johnson et al.) provide valuable clinical insights into complex disease management, emphasizing the need for integrated medical and surgical approaches. Ford at al., found that detection of peripheral blood smear bacteremia in clinically ill small animals is associated with a high in-hospital mortality in this study and supports the importance of timely diagnostics and consideration of early intervention.

## Zoonotic threats and surveillance

The growing concern over zoonoses is addressed through comprehensive surveys, such as the investigation performed by Mazzotta et al., on emerging and neglected pathogens in dog and cat shelters in North-East Italy, which highlights the need for continuous monitoring and updated risk mitigation strategies. Similarly, the characterization of tuberculosis granulomas in alpacas (Agulló-Ros et al.) not only advances our understanding of disease pathology in camelids but also underscores their potential role in zoonotic transmission.

## Future directions

Collectively, these contributions illustrate the dynamic nature of veterinary pathology and its critical role in safeguarding animals and public health. Moving forward, interdisciplinary collaboration will be essential to integrate molecular diagnostics, AI-driven tools, and advanced imaging into routine practice. Addressing antimicrobial resistance, emerging pathogens, and complex disease syndromes will require sustained research efforts and global cooperation.

By presenting these innovative studies, this Research Topic aims to inspire further exploration and guide future strategies in veterinary pathology and ultimately enhance diagnostic accuracy, therapeutic efficacy, and zoonotic risk management.
